# Historical changes (1905-present) in catch size and composition reflect altering fisheries practices on a small Caribbean island

**DOI:** 10.1371/journal.pone.0217589

**Published:** 2019-06-13

**Authors:** Mark J. A. Vermeij, Kelly R. W. Latijnhouwers, Faisal Dilrosun, Valérie F. Chamberland, Caroline E. Dubé, Gerard Van Buurt, Adolphe O. Debrot

**Affiliations:** 1 Carmabi Foundation, Willemstad, Curaçao; 2 Aquatic Microbiology, Institute for Biodiversity and Ecosystem Dynamics, University of Amsterdam, Amsterdam, the Netherlands; 3 Department of Agriculture and Fisheries, Ministry of Health, Environment and Nature, Willemstad, Curaçao; 4 Independent researcher, Willemstad, Curaçao; 5 Wageningen Marine Research, Den Helder, the Netherlands; Department of Agriculture and Water Resources, AUSTRALIA

## Abstract

Effective assessments of the status of Caribbean fish communities require historical baselines to adequately understand how much fish communities have changed through time. To identify such changes and their causes, we compiled a historical overview using data collected at the beginning (1905–1908), middle (1958–1965) and end (1984–2016) of the 20^th^ century, of the artisanal fishing practices and their effects on fish populations around Curaçao, a small island in the southern Caribbean. We documented historical trends in total catch, species composition, and catch sizes per fisher per month for different types of fisheries and related these to technological and environmental changes affecting the island’s fisheries and fish communities. We found that since 1905, fishers targeted species increasingly farther from shore after species occurring closer to shore had become rare. This resulted in surprisingly similar catches in terms of weight, but not composition. Large predatory reef fishes living close to shore (e.g., large Epinephelid species) had virtually disappeared from catches around the mid-20^th^ century, questioning the use of data from this period as baseline data for modern day fish assessments. Secondly, we compared fish landings to *in-situ* counts from 1969 to estimate the relative contributions of habitat destruction and overfishing to the changes in fish abundance around Curaçao. The decline in coral dominated reef communities corresponded to a concurrent decrease in the abundance and diversity of smaller reef fish species not targeted by fishers, suggesting habitat loss, in addition to fishing, caused the observed declines in reef fish abundance around Curaçao.

## Introduction

Historical accounts by the earliest European explorers of Caribbean waters report large abundances of manatees, large sharks, sea turtles and monk seals that are unimaginable given the current state of these ecosystems (e.g., [1–3]). The unprecedented depletion of nearshore marine life around Caribbean islands is arguably best illustrated by the changes in their fish communities. Unsustainable human exploitation has resulted in present day fish communities that differ markedly in composition and abundance from fish communities observed only decades ago [2, 4–8]. Larger (predatory) fish have become especially rare and no longer affect other reef community members through behavioral and trophic interactions, a phenomenon referred to as “ecological extinction” [9, 10]. The disappearance of large fish has been linked to an increase in former prey species (prey release) [10, 11], including those that can feed on or destroy living corals (e.g., [12, 13]), an increase in disease prevalence in fishes as infected hosts are no longer effectively culled [14] and reductions in reef accretion due to the historical overfishing of parrotfishes [15]. Therefore, over-exploitation of (predatory) fishes has resulted in cascading effects that have affected the functioning of reef ecosystems as a whole [9, 16, 17].

However, while historical changes in fish community composition have had negative consequences for Caribbean reef systems, we generally have limited information on the magnitude of these changes as many Caribbean fish communities had already been impacted (e.g., [5]) before systematic monitoring of catch data began in the 1950s [18, 19] and *in-situ* monitoring 30 years later [20, 21]. This example of the “shifting baseline syndrome”, whereby ecological change is wrongly assessed by using inappropriate baselines, obviously leads to a severe underestimation of the magnitude of changes in fish stock sizes through time [3, 6], that often takes the form of denial that such changes even occurred (e.g., [22–24]).

Fish communities on the Caribbean island of Curaçao have been fished for millennia [25]. European explorers arrived in the 16^th^ century and encountered an indigenous population for whom fishing was a valuable source of nutrition, possibly already since 2500 BC [26]. Descriptions of the first ‘professional’ artisanal fisheries (i.e. people that engaged in fisheries to generate income) date from 1824 [27]. On Curaçao, a former Dutch colony in the southern Caribbean, a qualitative and quantitative overview of the Curaçao fishing industry was undertaken by Boeke in 1905 [28] and in 1908 by Breeman [29]. These quantitative descriptions of fish landings predate most existing descriptions of “historical” Caribbean fisheries by nearly half a century (e.g., [4, 18, 21]). Comparing fish landings from 1955 [29] to those of 1905 [28] already shows that fish species commonly landed in 1905 such as Nassau groupers (*Epinephelus striatus*), king mackerel (*Scomberomorus cavalla*), blue marlins (*Makaira nigricans*), and ocean triggerfish (*Canthidermis sufflamen*) had already become rare or absent in landings in 1955.

Here, we review the changes in Curaçao fish communities spanning the entire 20^th^ century. We used catch data from the beginning [28, 29], the middle [29–31] and end of the century (e.g., [32–37]) to analyze changes in Curaçao fish communities based on fish catch characteristics and changes in fishing practices in terms of methods and number of people involved. Observed changes in reef fish community composition were also compared to two fisheries-independent data sets describing the island-wide abundance of fishes targeted and not targeted by fishing in 1969 and 2011 [31, 33]. The data from 1969 predates the dramatic decline in coral cover that started in the late 1970s and the catastrophic die off of the herbivorous sea urchin *Diadema antillarum* in 1983 [20]. Because data on fishing practices on Caribbean islands from the first half of the 20^th^ century are scarce, abovementioned studies provide an opportunity to reconstruct the effects of fishing and its effects on fish communities in Curaçao and elucidate the factors likely responsible for these changes.

### Data analysis & methods

To reconstruct the abundance and composition of historical fish communities, we first analyzed catch data from several historical and recent reports on the composition of fish landings on Curaçao (12°N 69°W) by fishers that fished, generally by boat, with the purpose of selling their catches, i.e., not subsistence fishers that primarily fished to feed family and relatives (Table 1). Additionally, based on the same resources, a brief overview of the characteristics of the island’s fishing industry from the early 1900s until now is provided (Table 2).

**Table 1:** Overview of main data sources used to reconstruct the fishing industry in Curaçao through time (1905-present).

Study	Time period	Fishing method	Unit	Data
[28] Boeke (1907)	April 1905 -November 1905	Handline/trolling	numbers, species identity	Landings of individual fish (n = 133,392) at the fish market of Curaçao’s capital city. Counts on fleet composition and number of fishers
Data from Dr. P.J. van Breeman in: [29]	January 1908—December 1908	Handline/trolling	numbers, species identity, total catch (in tons)	Landings of individual fish (n = 1,265,486) and catch (in ton per year) per fisher (n = 498). Counts on fleet composition and number of fishers.
Data from Dr. G.C. Salmon in: [29]	January 1958—December 1958	Handline/trolling	total catch (in tons per year), numbers, species identity, average catch per fisher (in tons per year)	total catch and average catch per fisher
[30] Kristensen (1965)	January 1965—December 1965	Handline/trolling	total catch (in tons)	Total weight of all landings and estimates of fleet composition and number of fishers
[7] Van Buurt (2001)	1994–2001	Handline/ trolling	total catch (in tons per year), general catch composition.	Total weight of all landings and estimates of fleet composition, location of fishing activity (i.e., near- vs offshore) and number of fishers
[34] Waitt Institute (2016b)	January 2016—June 2016	Handline/trolling	numbers, species identity, total catch per species (in kgs)	Catch data from surveys at 14 fish landing locations, fishers’ average daily catch (in kg) and the fisher’s average number of fishing days per week
[29] Zaneveld (1962)	February 1955—April 1955	Fishtrap	numbers, species identity	Landings of individual fish (n = 1,192) collected from two sites: Bullenbaai (18 fishtraps surveyed over 36 days) and Caracasbaai (31 fishtraps surveyed over 93 days). Known soak times.
[36] Schultink and Lindenbergh (2006)	February 2006—May 2006	Fishtrap	species identity, number, individual weight	Landings of individual fish (n = 773) collected from five sites. 17 fish trap catches analyzed. Known soak times.
[35] Johnson (2010)	May 2008—August 2008	Fishtrap	species identity, number, individual weight	*In-situ* observations of trap catches (n = 1,028) collected from three sites. 190 fishtrap catches analyzed. Known soak times.
[34] Debrot (2013)	1950, 1954–1970, 1997–2005	Spearfishing	numbers, species identity, average weight per species	Data derived from pictures; 1950: 10 catches, 1954–1970: 20 catches, 1997–2005: 21 illegal catches.
[26] Zaneveld (1962)	1956–1957	Spearfishing	numbers, species identity, total catch per species (in kgs)	Landings of individual fish (1956: n = 55, 1957: n = 119) collected during two spearfishing tournaments lasting 5 hrs in which 22 (1956) and 21 (1957) fishers took part
[31] Nagelkerken (1970)	1969	*In-situ* fish counts	density of shallow water reef fishes	Fish counts on 22 sites along the south coast of Curaçao in 4x4 quadrats.
[33] Chamberland (2011)	May 2011—November 2011	*In-situ* fish counts	density of shallow water reef fishes	Fish counts on 22 sites along the south coast of Curaçao in 4x4 quadrats.

**Table 2:** Overview of the number of fishers, total annual catches and size and composition of the fishing fleet on Curaçao between 1905 and 2016.

Year	Fishers	Fishing boats	Total annual catch	Composition of fleet			
	(total n)	(total n)	(in tons)	*Canoa*	Sailboat	Rowboat	Motorized boat
1905	1118*	311	1878*	300	0	7	4
1908	498	162	1245				
1959	652	332	750	40	27	217	48
1965	600	100	1100				
1984	450	145	944				
1998	390	255	900				
2001	390**	262	1050				
2016	183	239	692	0	0	32	207

* This number appears high because Boeke [25] also included all persons qualifying themselves as “seaman” as part-time fishers. The number of full-time fishers is 46 and we used the ratio of full to part-time fishermen in 1908 (1:3) to calculate the total number of fishers in 1905 (n = 184) and used this number in all calculations.

** The number of fishers reported for 2001 appeared low (n = 155) and we used the number for 1998 instead.

### Catches with lines

Catch data from 1905 were originally collected in numbers of individuals per species, whereas later catches were expressed in kilograms. To compare these datasets, catches expressed in numbers of individuals were transformed to kilograms by multiplying the number of fish belonging to a species by that species’ oldest reported average weight of an individual fish from Fishbase [38]. Because we assumed that weight-length relationships did not significantly change between 1905 and 1950 (the year with the oldest available size data), we could have underestimated the size of catches in 1905 if average fish sizes had declined through time. However, because fishing lines were foremost made of cotton or linen during the first half of the 20^th^ century, they often broke when larger (pelagic) fish were hooked reducing the potential for changes in size selectivity of the fisheries during this period. Comparisons of catch per unit effort (CPUE) estimates require corrections to account for the efficiency of changing fishing practices through time, especially for handline or boat-based fishing practices [39]. Estimates of CPUE on Curaçao through time are also problematic to derive due to (1) changes in the efficiency of the fleet through time due to technological advances (e.g., fish finders, outboard engines, introduction of nylon lines); (2) fishing effort shifting to previously non-targeted species resulting in (temporarily) higher catches; and (3) environmental changes that can also contribute to changes in the size of local fish stocks (e.g., habitat loss [40]). Changes in catch sizes through time were therefore based on the average catch (kg) per fisher per month (CFM), also because catch data were sometimes collected for several months in a year only (Table 1). No spawning aggregations are mentioned in any of the sources we used (Table 1) and therefore aggregations were unlikely to have affected catch data comparisons. The weather is calm year-round in Curaçao and therefore also not expected to be factor confounding comparisons catch sizes among years as calm months could have been compared with rough months in other years. CFM was then used as an abundance index of each component of the fish community that is vulnerable to fishing without considering the effects of changing fishing practices. The fact that studies only collected data as total catches for all fishers combined for certain periods of the year also precluded the calculation of variance or error estimates for individual years, especially in studies from the first half of the 20^th^ century.

### Catches with traps

Catches from fish traps (locally known as ‘kanasters’, see [35] for a description) from 1955, 2006 and 2008 were derived from [29], and [35, 36], respectively (Table 1). In order to compare the 1955 dataset with the one from 2006 and 2008, the weight of the total catch per species was again calculated from size frequency data using length-weight relationships from FishBase as described above. Because the design and size of fish traps has changed little through time (see below), we inventoried fish trap catches through time (1955 to 2008) to more reliably estimate changes in CPUE and fish abundance. All fish trap data were expressed as kg of fish caught per fish trap per 24 hours soak time (i.e., time that traps were placed on the reef) as a measure of CPUE. For each surveyed year, the total catch (kg) and the total catch per species were calculated as averages only, as total catches and number of fishing days were the only data provided in older studies, again precluding the calculation of variance metrics.

### Catches with spear guns

Data from spearfish catches from 1950, 1954–1970, and 1997–2005 (Table 1) were used to assess changes in the number and identity of larger reef-associated fish that are usually targeted by spearfishers (e.g., *serranidae*) and originate from previously unpublished data by Debrot [37], on the size, number and identity of fishes from pictures taken during spearfishing tournaments (1950), individual spearfishing trips (1954–1970) and, more recently, of illegal spearfishing catches (1997–2005). Data from annual spearfishing tournaments likely represent relatively smaller catches compared to fishers pursuing maximum fish yield immediately before and after these years. Size estimates were derived by comparing fish lengths to objects and persons of known lengths in the photographs. Data from 1956 and 1957 were collected during spearfishing tournaments during which large fish were targeted and published by Zaneveld [29]. All data after 1976 [37, 41] were derived from visual surveys of illegal catches as spearfishing became illegal on Curaçao in 1976. The CPUE (mean ± SE) of spearfishers’ catches was calculated as the catch weight (kg) per spearfisher per hour for the total catch and that of individual fish species.

### Fishes not targeted by fishing

*In-situ* reef fish counts were analyzed at two time points (1969 and 2011) to assess the potential contribution of habitat loss on changes in fish abundance. Habitat loss (“reef degradation”) has occurred Caribbean-wide since the late 1970s, with mass-mortalities of the key reef-building *Acropora* species, the massive die-off of the keystone grazing species *D*. *antillarum* and repetitive coral bleaching events [20]. Nagelkerken [31] studied the abundance of reef fishes in *Acropora* fields in 1969 along the south coast of Curaçao using rotenone. He counted the density of individual fish species in 4x4 quadrats between 2 and 5 meters depth at 16 sites. In 2011, 9 of the 16 sites surveyed by Nagelkerken were revisited and visually surveyed [33]. Data from both survey periods were converted to average fish density per m^2^ (mean ± SE) for historically abundant fish species (arbitrarily defined as those having >20 total individuals observed in 1969). In addition, cryptic species (i.e., blennies, brotulas, spaghetti eels) were also excluded to minimize the probability that observed differences between years resulted from the different methodologies (rotenone vs visual surveys) rather than the actual changes in fish abundance through time. For small fish species that are not targeted by fishers and that have a strong affinity for habitats dominated by live coral (e.g., Pomacentridae, Pempheridae), we assumed that the changes in their abundance are foremost the result of ecological changes that have occurred on Curaçaoan reefs between 1969 and 2011, in the form of prey release or changes in habitat availability.

## Results

### Short historical overview of Curaçaoan fishing practices

Fishing around Curaçao mainly occurs in the form of traditional artisanal fisheries that can be broadly categorized as (1) bottom fishing that primarily target demersal species (i.e. reef-associated species) up to a depth of 200 m using handlines and (2) trolling fisheries targeting nearshore pelagics using handlines dragged behind a boat [42]. The first report of handlining dates from 1824 by Teenstra [27] and since then handlining (bottom fishing and trolling) has accounted for the majority (~85%) of demersal and pelagic fish landings [7, 18, 28, 29]. Around 1900, the most commonly used fishing vessel was a small canoe (*canoa*), often with a small sail (Table 2) with room for only one or two fishers and limited space for storing larger fish species [28]. Due to the small size of their boats, fishers focused on reef-associated species and only a few fishers (11 in total) ventured out to the open sea to target nearshore or migratory pelagic species, but stayed within view of the island [28]. Fishers would return to shore to land their catches (3–4 times a day [28]) and generally fished from 0500 to 1200 AM [29]. Because at the beginning of the 20^th^ century fishing lines were made of cotton or linen, they often broke when larger (pelagic) fish were hooked. Such fish became more intensively targeted after the introduction of nylon fishing lines around 1934 [43].

Because many people preferred working for the growing oil industry with more lucrative wages [29], the number of fishers and fishing boats remained relatively constant (~600 and ~300, respectively) between 1904 and 1965 despite a 4-fold increase in Curaçao’s population size. In 1959 most fishermen (385 out of 652) fished part-time, mostly (217) from rowing boats (locally known as a *jola* or *vlet*) and only 48 fishers used motorized fishing vessels [29]. The use of motorized vessels increased after the middle of the 20^th^ century and is 86% at present [44], though the number of functional fishing boats is likely half that of the total number of fishing boats present on the island [34]. The introduction of larger motorized boats and ice during the last quarter of the 20^th^ century allowed fishers to go further and stay out longer allowing the targeting of larger fishes, including pelagic species. More industrial forms of fishing, such as trawling (around 1955) and longlining (around 2000) were attempted, but proved unsuccessful and were stopped within a few years [18, 29]. Presently, motorized boats smaller than 5 meters and equipped with an outboard engine (average engine power: 66, range: 2.5–230 horsepower) are the most commonly used type of full-time fishing vessel. Most fishers (71%) fish part-time and fishing is the main source of income for a third of all fishers (31%) [34]. While fishing contributed ~4% to the Curaçao’s Gross Domestic Product in 1904 [42], it has decreased to less than 0.001% in 2015 due to the low import prices for foreign fish and the decreasing CPUE which makes fishing largely unprofitable among other factors [45].

Recently emerged tourist charter vessels focus on catch and release of mostly nearshore pelagic species and are therefore considered a negligible factor driving potential overfishing on the island [18]. The use of gill nets was always limited, but increased during the first part of the 20^th^ century; 6 fishers (1.2% of total) used nets in 1905 whereas 25 fishers used nets in 1959 (3.8%). Most “net fishers” fished from beaches to which they often had exclusive rights to fish [46]. Specific seine nets (*redas*) are used to this day to catch schools of bigeye scads (*Selar crumenophthalmus*) that migrate along the island’s coast. The use of all nets targeting reef fish, except *redas*, was made illegal in 2014, though illegal gillnetting for reef species still occurs and is poorly regarded by the large majority of local fishers for its high bycatch rate [47].

Antillean chevron shaped fish traps made of galvanized chicken wire were introduced in the late 1800s as a new method for demersal (reef) fishing [28, 48]. Fish traps are handmade and while they differ slightly in exact dimensions, they all have a volume of ~0.5 m^3^ (mesh size: 2 to 6 cm) and have remained largely unchanged from the late 1800s until now [28, 35, 48]. The advent of recreational diving in the 1960s resulted in a decline in trap fishing as recreational divers often damaged fish traps to release trapped fish. In the early 1990s less than 10% of all fishers regularly used fish traps [49]. A law was passed in 2009 forcing fishers to equip their traps with escape gaps to reduce bycatch of smaller fish species [35]. Hawaiian slings were introduced in the 1940s and immediately became a very popular fishing method in Curaçao [50]. In the 1950s spear guns followed. Using Hawaiian slings first, and later spear guns, spearfishers foremost targeted large reef-associated piscivores such as groupers (Serranidae), jacks (Carangidae), rays and sharks (Elasmobranchii) [7] that have become extremely rare on present day Curaçao reefs [51]. By the late 1960s, it was already evident that spearfishing had led to overfishing of especially large predatory fishes and spearfishing was made illegal in 1976 [7, 42]. Enforcement of fishing regulations has, however, been largely ineffective [52].

### Handline fisheries

**Fig 1:**
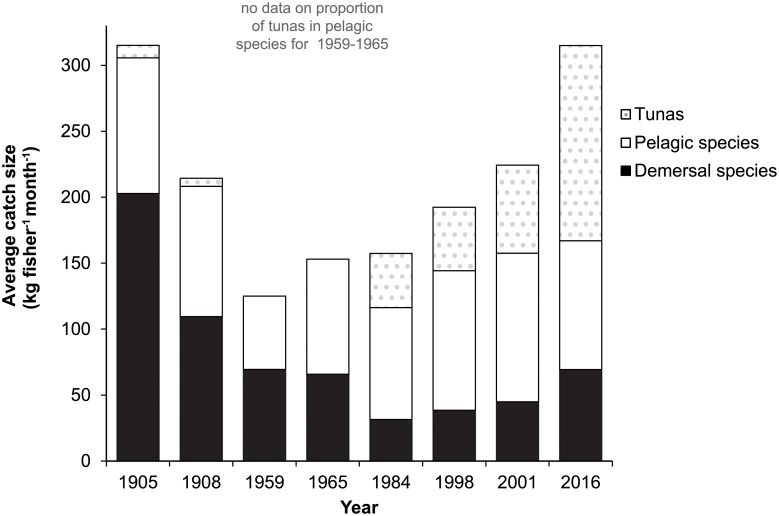
The average weight (in kg) of fish caught per fisher per month. Demersal (or reef-associated) species are all species caught directly over or near Curaçao’s fringing reefs, whereas pelagic species are caught off-shore. The proportion of pelagic catches comprised of tuna species is shown separately for all years except 1959 and 1965.

The average CFM declined by 60% from the beginning (1905: CFM = 313) to the middle of the 20^th^ century (1959: CFM = 115), after which the CFM increased again over the second half of that century to that reported at the beginning of the century (2016: CFM = 315) (Fig 1). Despite increased fishing efficiency, the proportion of the total catch comprised of large reef-associated species has decreased 3-fold (Fig 1). During the first half of the 20^th^ century the proportion of the total catch comprised of pelagic species other than tuna increased, while catches of reef-associated species declined. In the mid-1980s, catches of these pelagic species started to decrease and the proportion of the total catch comprised of tuna, which was negligible until then, started to increase. Combined, a pattern emerges whereby the reduction in one type of fishes targeted by fishers is compensated by targeting a new, historically harder-to-access group of fishes increasingly farther off-shore (i.e., from reef-associated species, to nearshore pelagics and then to offshore pelagics). The estimated total annual catch declined over time from nearly 2000 metric tons at the beginning of the 20^th^ century to less than metric 1000 tons at present (Table 2).

**Fig 2:**
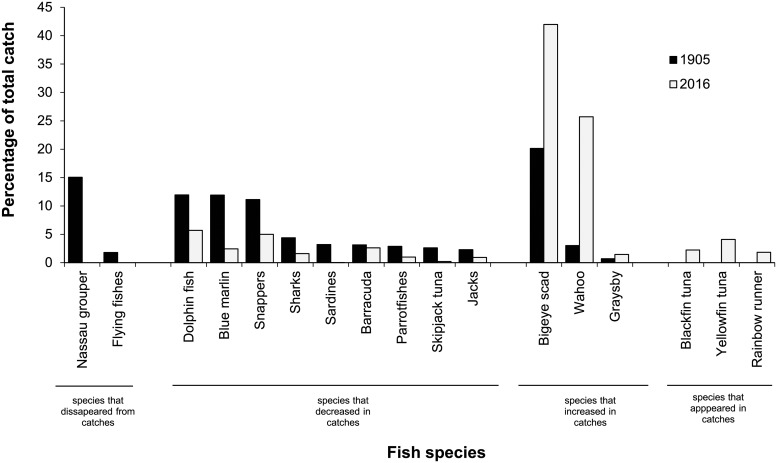
Species-specific differences in composition (based on weight) of handline catches between 1905 and 2016. Only species that accounted for >1% to the total catch in 1905 were included. Species were grouped in four categories: species that disappeared, appeared, increased or decreased from catches between 1905 and 2016.

Significant differences were observed when the species composition of handline catches from 1905 to 2016 were compared for fish species accounting for >1% of the weight of the total catch (Chi-Square = 211.0, df = 11, p< 0.001, Fig 2). The proportion of some species such as dolphinfish (*Corryphaena hippurus* and *C*. *equiselis*) and blue marlin (*Makaira nigricans*) decreased on average by 64.8% (n = 9 species, SD 21.3, Fig 2). Similarly, some species like Nassau grouper disappeared from present-day catches while the proportional catch other species like wahoo (*Acanthocybium solandri*) and grasby (*Cephalopholis cruentata*) increased on average by 63.7% (n = 3 species, SD 21.3, Fig 2). Certain species that were historically not targeted, such as some tuna species, only recently appeared in landings (Fig 1).

### Fish traps

**Fig 3:**
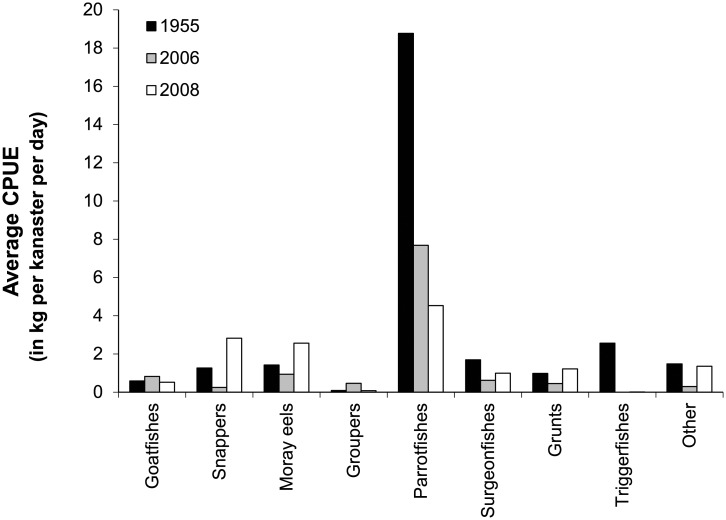
The changes in average trap catches between 1955 and 2008 per unit effort (CPUE; in kg fish caught per kanaster per 24 h soak time). Averages are calculated from total catches and number of days that kanasters were placed on the reef.

The average CPUE for fish traps declined 46% between 1955 and 2008, i.e., from 28.9 kg trap^-1^ d^-1^ in 1955, down to 15.5 in 2008. The composition of catches also significantly changed over the same time period (Fig 3, Chi-Square = 16.5, df = 8 p< 0.05). Small parrotfish species dominated fish trap catches through time on Curaçao despite an overall 4.2-fold reduction in landings (in kg) between 1955 and 2008. The proportion of other fish groups in fish trap catches also decreased, especially of Balistidae (500-fold), and to lesser degree Acanthuridae (2-fold). The relative proportion of Lutjanidae and Muraenidae in trap catches increased (2-fold) in 2008 compared to 1955. Overall, the CPUE of fish trap fishing targeting ‘small reef fishes’ decreased between 1955 and 2008 with, similar to line-fishing, noticeable changes in catch composition.

### Spearfishing

**Fig 4:**
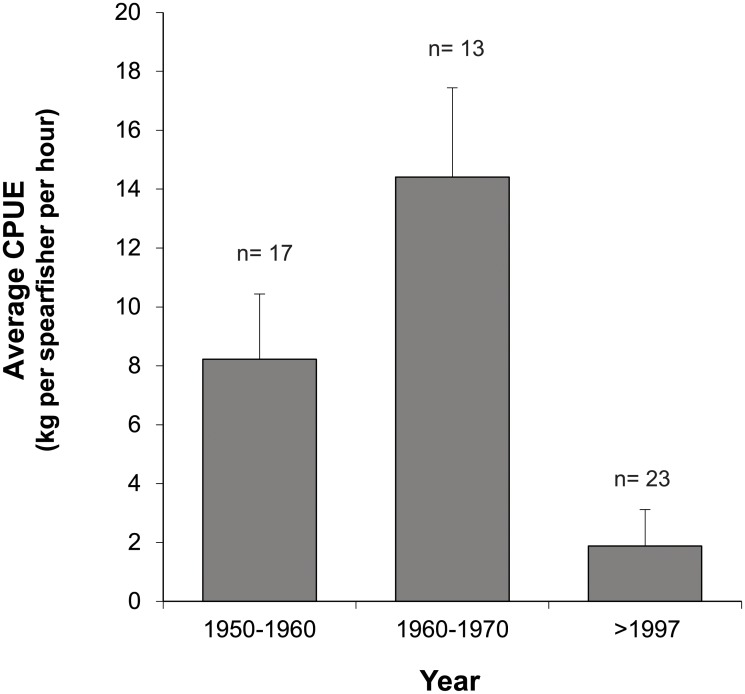
The changes in spearfish catches for the second half of the 20^th^ century per unit effort (CPUE; in kg fish caught per spearfisher per hour). No data available for catches between 1970 and 1997. Error bars represent standard errors.

**Fig 5:**
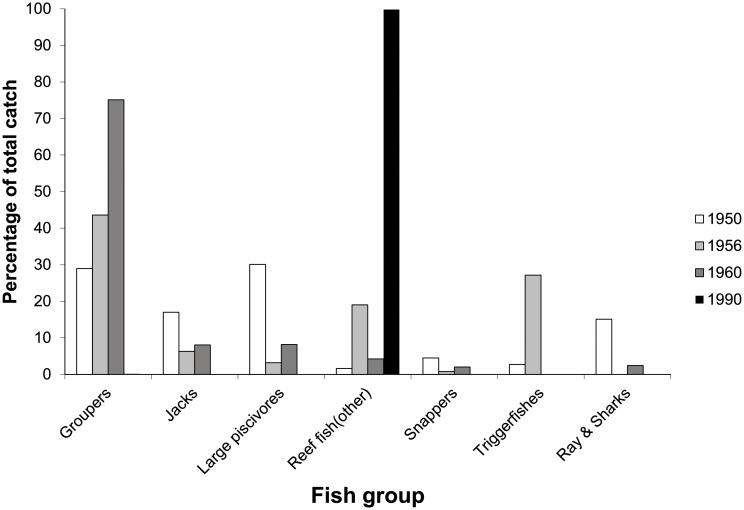
Changes in the composition of spearfish catches over the second half of the 20^th^ century. Note that many bars are not visible because values are zero.

The average catch of larger reef fishes caught with spear guns declined by 77% between 1950 (8.2 kg fish fisher^-1^hour^-1^) and the end of the century (1.9 kg fish fisher^-1^hour^-1^) (Fig 4). Similar to handline and fish trap catches, a significant change in the composition of (landed) fishes caught with spear guns occurred between 1950 and 1990 (Fig 5, Chi-Square = 6032.4, df = 6, p< 0.001). Whereas groupers, jacks, barracudas, sharks and rays comprised >80% of the catch (in kg) in 1950, the catch in 1990 was entirely (99.8%) comprised of parrotfish and small reef fishes (i.e., members of the families Serranidae, Carangidae and Acanthuridae).

### *In-situ* comparisons of fish communities through time

**Fig 6:**
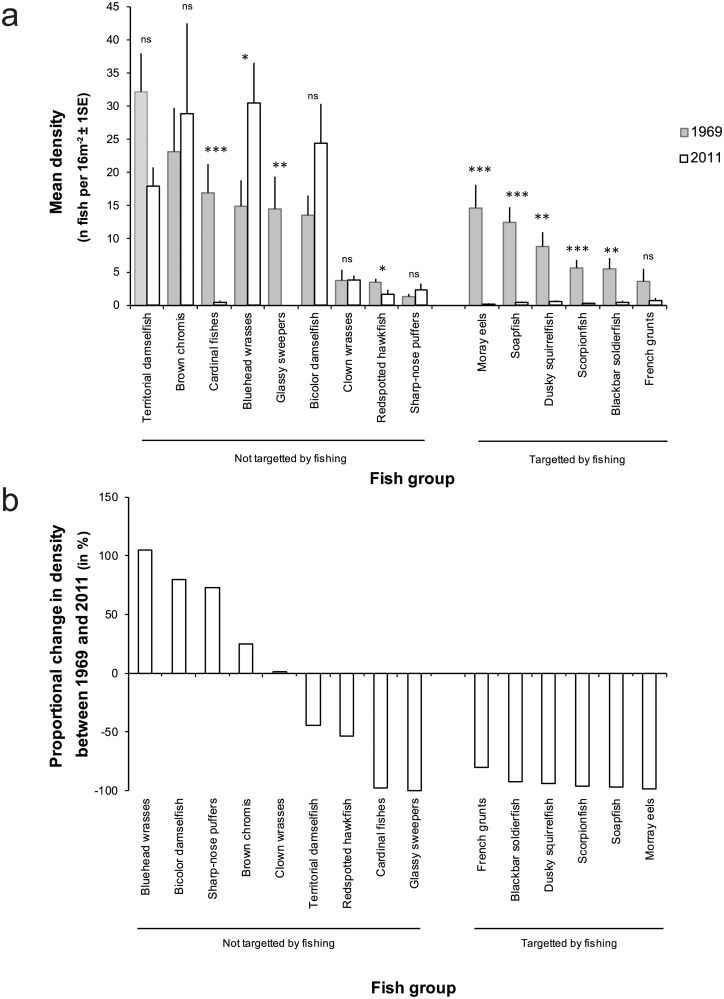
**(a) Differences in the *in-situ* density of fish species targeted and not targeted by fishers between 1969 and 2011**. To better illustrate the changes in density independent of species-specific differences in abundance, the proportional change in the density of each species is also shown in (b).

Between 1969 and 2011, the abundance of non-cryptic fish groups or species not targeted by fishers either increased (bluehead wrasses (*Thalassoma bifasciatum*)), did not change {e.g., brown chromis (*Chromis multilineata*); bicolor damselfish, (*Stegastes partitus*)} or decreased in abundance {redspotted hawkfish (*Amblycirrhitus pinos*); cardinal fishes (Apogonidae), glassy sweepers (Pempheridae)} (Fig 6a). Overall, the abundance of reef-associated fish targeted by fishing decreased more dramatically than that of fish groups that were not actively fished, i.e., on average 93% (SD: 7, n = 6) versus 1% (SD: 77, n = 9), respectively (Fig 6b). The direction in which the abundance of fishes not targeted by fishing changed appeared loosely related to habitat preference based on [38]. Generally, fish species that move around in the water column directly above the reef during the day did not change (clown wrasses) or increased in abundance through time (bicolor damselfish, chromis, bluehead wrasses and puffers), whereas more stationary species that live closely associated to or on the framework built by corals (territorial damselfishes, cardinal fishes, sweepers, hawkfish) decreased in abundance.

## Discussion

Marine species in the waters around Curaçao, adjacent islands and the rest of the Caribbean have been subject to exploitation by humans for millennia [2, 25, 53]. Even in pre-Columbian times, exploitation of marine organisms, especially large fishes and turtles, was already unsustainable on several Caribbean islands [5], but it was not until after the arrival of Europeans in the region that increasing human population size and technological developments translated into major overfishing, particularly during the 20^th^ century (e.g., [3, 54, 55]).

Using data on historical catches from Curaçao spanning the entire 20^th^ century, we found that fishers at the beginning of the 20^th^ century targeted reef fishes near shore, but started to target near-shore pelagic species (e.g., wahoo, common dolphin fish) in the mid-20^th^ century after large reef fishes had decreased in abundance. While boat-based fishers moved off-shore, spearfishers, that usually swim, and thus cannot move far offshore started targeting smaller and less desirable reef-associated fish species once large, predatory reef fish species had declined in abundance (Fig 5). A second shift in the composition of catches occurred towards the end of the century, when fishers shifted from near-shore pelagics to off-shore pelagic species, especially tunas that are mostly caught at night around floating oil tankers. The shift towards more pelagic species during the 20^th^ century was facilitated by technological improvements whereby canoes were replaced by larger boats (Table 2) that could go farther off-shore [28, 29], which in turn were replaced by motorized boats after the 1960s [7].

While (historical) catch data are inherently hard to compare due to differences in methodology and data recorded, the average size of handline catches (in *kg fisherman*^*-1*^ *month*^*-1*^) were very similar in 1905 and 2016. Catches between 1959 and 2011 were smaller compared to from those from the beginning and end of the 20^th^ century (Fig 1). However, using total weight to compare landings through time (e.g., between 1905 and 2016), ignores the large changes in the composition of these landings indicating that fishers shifted from reef-associated to pelagic species, fishing increasingly farther off-shore through time (Fig 1). This phenomenon is a community (from nearshore to offshore fish communities) rather a species-level version of “sequential overfishing”, whereby local declines in catches are compensated by expanding the area over which species are harvested, resulting in similar catch weights through time [56].

Using only the weight of total catches severely masks co-occurring changes in the composition of fish landings that would signal overfishing has occurred for previously targeted fish species. For example, Nassau groupers, one of the most caught fish in 1904 (Fig 2), had completely disappeared in landings by 2016, a change that is not reflected in total catch weights; increased wahoo catches compensated for volumetric reductions from Nassau grouper. Technological advancements (e.g., nylon fishing lines, outboard engines) resulting in higher “fishing power” or CPUE [40], shifts to historically untargeted fish species such as tuna and the emergence of a small number of very active, skilled fishers accounting for the majority of the total catch [34] further complicate straightforward comparisons of catch data through time based on total weights alone. As a result, characteristics of catches based on total weight alone do not reflect the changes in the abundance or community composition of fishes around Curaçao and consequently do not produce the information needed to assess or manage the island’s fish communities or even worse, suggest that no problem requiring management interventions exists at all.

If only handline catches for the second half of the 20^th^ century are considered, the average catch size increased more than two-fold (Fig 1). However, based on catch data from fish traps and spearfishing, a decline in total catch size of 46% (Fig 3) and 77%, respectively, (Fig 4) occurred over the same period. Consequently an assessment of the true changes in fish communities are best based on fishing methods that have remained relatively similar through time such as fish trap and spearfishing that have not seen major technological improvements.

Due to technological improvements such as the introduction of nylon fishing lines in 1934 and the use of larger and faster fishing vessels in the first half of the 20^th^ century [7, 28, 29], we would expect catches around 1960 to be higher than in 1904. However, despite improved fishing efficiency, the total catch decreased by 60% over this 50-year period (Fig 1), indicating that the first effects of fishing were already evident in the mid-20^th^ century. Large predatory fishes such as Nassau groupers, king mackerels and cubera snappers (*Lutjanus cyanopterus*) that were commonly caught at the beginning of the century had completely disappeared from the catches 50 years later [29]. The removal of large predatory fish likely came with consequences for the rest of the ecosystem. For instance, concomitant to the disappearance of Nassau grouper from recorded catches, smaller grouper species such as coneys (*Cephalopholis fulva*) and graysbys (*C*. *cruentata*) became released from competition with larger grouper species and more than doubled in abundance, despite being fished [51]. Predators also no longer reduced the abundance of the threespot damselfish (*Stegastes planifrons*) that create algal gardens at the expense of live coral colonies [12]. Both examples illustrate how the removal of predatory fish species affects the abundance of smaller reef fishes and therefore indirectly the ecological processes shaping reef communities.

Using data from the mid-20^th^ century as a historical baseline for Curaçao’s present fish populations would lead to a severe underestimation of the actual changes in fish communities that started 50 years prior. Even the 1900s data may not provide an appropriate historical baseline for the Curaçao fish populations given the fact that other animals hunted by humans such as monk seals and manatees had already disappeared from the island’s marine ecosystems by then [57, 58]. Fish accounted for 8% of the entire value of all goods imported to Curaçao in 1905 [28]. The proportion of locally consumed fish that was imported was 66% in 1955 [29] and is currently described as “significant” [18]. In none of the historic resources used for this study did we find any evidence for export of fishes caught by artisanal fishers. Combined, these data strongly suggest that local demand for fish could not be met by local catches at the beginning of the 20^th^ century. Therefore, there is a need for an appropriate baseline to guide fishing and reef management efforts [59]. Cognizant of the “shifting baseline” problem in fisheries [6], our results clearly show that major changes occurred prior to the advent of quantitative coral reef science in the 1950s and that using data from this period for baseline purposes carries inherent limitations to understanding the historical, ecological changes that have taken place in Caribbean reef fish communities.

The abundance of fish groups or species not targeted by fishers between 1969 and 2011 either increased, remained the same or decreased depending on species (Fig 6). The increase of species such as blue head wrasses and bicolor damselfish through time likely stems from prey release [16] whereby decreases in the biomass of fishes at a higher trophic level (i.e., predatory fishes) caused increases in the prey biomass at the next lower trophic level [16]. Certain fishes not targeted by fishing also declined in abundance, but involved mainly species dependent on live coral or the structures it produces. The loss of coral cover around Curaçao due to disease, coastal development, pollution, bleaching and storms over the last few decades [60, 61] undoubtedly affected the abundance of such obligate reef-associated fish species illustrating the role of habitat degradation driving the declining abundance of at least certain reef fishes in addition to fishing. Other forms of habitat degradation could also have contributed to the decline of fishes in general. For example, Debrot et al. [62] documented significant habitat deterioration of seagrasses and mangroves in one of the largest inland bays on Curaçao (Spanish Water), which is the most important nursery habitat for many fishes on the island [63]. Confirming earlier studies relating reef complexity and the abundance of reef-associated fishes [21, 64, 65] and species other than fish [66], this example is only serves to illustrate that in addition to fishing, several forms of habitat degradation also contribute to the observed declines of at least certain fish species.

## Conclusions

The average CFM has remained surprisingly constant over the last century on Curaçao when considering landing sizes for handline fisheries. However, information in total landings, as volumes, proved to be misleading as fishers targeted new species through time after earlier targeted species had become rare, a phenomenon known as “sequential overfishing”. Total landings seem therefore only appropriate for species-specific fisheries (e.g., herrings, sardines) whereby fishers do not compensate losses in one species by shifting to others (e.g. spearfishers) or by expanding the area where harvesting takes place (e.g., line fishing). Our study demonstrates how understanding the historical changes in fish community structure clearly requires a context broader than fishing alone, given the decline in certain reef fishes such as cardinal and hawkfishes that are not targeted by fishing, but with a strong dependence on live coral which has decreased enormously in the Caribbean over the last decades. This information is important when designing management strategies on small islands like Curaçao, because the amount of local support for such actions increases as persons or processes responsible for an undesired decline in marine resource (such as reef fish) are correctly identified. Fishers are probably more likely to support restrictive management action (i.e., local no-take zones) as they often feel they are singled out and accused of being solely responsible for decreases in fish abundance. New and existing regulations aimed at improving the health of Curaçaoan fish communities through land- and ocean-based regulations are, however, unlikely to achieve these improvements given the weak enforcement of fisheries regulations on Curaçao [34]. While certain fish species have declined almost solely due to overfishing (e.g., large grouper species), habitat degradation has resulted in the reduced abundance of especially obligate coral-associated fishes around small Caribbean islands such as Curaçao.
